# Overcoming Therapy Resistance in Colon Cancer by Drug Repurposing

**DOI:** 10.3390/cancers14092105

**Published:** 2022-04-23

**Authors:** Talal El Zarif, Marcel Yibirin, Diana De Oliveira-Gomes, Marc Machaalani, Rashad Nawfal, Gianfranco Bittar, Hisham F. Bahmad, Nizar Bitar

**Affiliations:** 1 Faculty of Medicine, Lebanese University, Beirut 1003, Lebanon; talalelzarif@gmail.com (T.E.Z.); m.machaalani@st.ul.edu.lb (M.M.); rn107@aub.edu.lb (R.N.); 2 Internal Medicine Residency Program, Department of Medicine, Boston University Medical Center, Boston, MA 02218, USA; marcel.yibirinwakim@bmc.org; 3 Department of Research, Foundation for Clinic, Public Health, and Epidemiological Research of Venezuela (FISPEVEN), Caracas 1050, Venezuela; dianacdeoliveirag@gmail.com; 4 Baylor St. Luke’s Medical Center, Houston, TX 770030, USA; gianfrancobittar@gmail.com; 5 The Arkadi M. Rywlin M.D. Department of Pathology and Laboratory Medicine, Mount Sinai Medical Center, Miami Beach, FL 33140, USA; 6 Head of Hematology-Oncology Division, Sahel General Hospital, Beirut 1002, Lebanon; nbitar@sahelhospital.com.lb; 7 President of the Lebanese Society of Medical Oncology (LSMO), Beirut 1003, Lebanon

**Keywords:** colorectal cancer, therapy resistance, drug repurposing, in silico drug screens

## Abstract

**Simple Summary:**

Despite improvements in standardized screening methods and the development of promising therapies for colorectal cancer (CRC), survival rates are still low. Drug repurposing offers an affordable solution to achieve new indications for previously approved drugs that could play a protagonist or adjuvant role in the treatment of CRC. In this review, we summarize the current data supporting drug repurposing as a feasible option for patients with CRC.

**Abstract:**

Colorectal cancer (CRC) is the third most common cancer in the world. Despite improvement in standardized screening methods and the development of promising therapies, the 5-year survival rates are as low as 10% in the metastatic setting. The increasing life expectancy of the general population, higher rates of obesity, poor diet, and comorbidities contribute to the increasing trends in incidence. Drug repurposing offers an affordable solution to achieve new indications for previously approved drugs that could play a protagonist or adjuvant role in the treatment of CRC with the advantage of treating underlying comorbidities and decreasing chemotherapy toxicity. This review elaborates on the current data that supports drug repurposing as a feasible option for patients with CRC with a focus on the evidence and mechanism of action promising repurposed candidates that are widely used, including but not limited to anti-malarial, anti-helminthic, anti-inflammatory, anti-hypertensive, anti-hyperlipidemic, and anti-diabetic agents.

## 1. Introduction

Colorectal cancer (CRC) is the third most prevalent cancer in the world, with more than 100,000 new cases and 50,000 deaths occurring in the United States during 2021 [1]. It is the third most common cancer in both sexes and the second most common cancer-related mortality cause [2]. It is projected that there will be an overall doubling of CRC cases in the following decades [3], with an estimated worldwide increase to 2.5 million new cases in 2035 [4]. In countries with a high human development index, the incidence and mortality rates of CRC have decreased predominantly in older adults, while in lower- and middle-income countries, mortality is increasing [2]. There is a growing incidence of CRC in younger groups, likely attributed to a high prevalence of certain risk factors such as poor diet, low physical activity, and higher rates of obesity [2].

Most CRCs arise from neoplastic polyps as stem cells progressively acquire genetic and epigenetic alterations [4]. Chromosomal instability, mismatch repair deficiency, and CpG hypermethylation underline the pathogenesis of CRC [5]. Significant molecular heterogeneity with altered mismatch repair mechanisms leading to microsatellite instability in *BRAF*-mutated sessile serrated adenomatous polyps and adenocarcinomas increase after 85 years of age [6]. Sporadic CRC accounts for 70% of new cases, usually following a specific succession of mutations in the adenomatous polyposis coli (*APC*) gene, followed by *KRAS*, *TP53*, and *DCC* mutations [7]. Familial cases correspond to approximately 25% of cases, and 5% occur in well-defined hereditary CRC syndromes such as familial adenomatous polyposis and Lynch syndrome [7].

The stage of the CRC at the time of diagnosis is crucial in determining survival. While the 5-year survival is approximately 90% for patients with stage I disease, in stage IV it decreases to less than 10%, highlighting the importance of early detection [8]. For localized early-stage CRC, surgery is the standard therapy [5]. In patients with stage II CRC with a high risk of recurrence after surgery or stage III disease (lymph node metastases), adjuvant chemotherapy using oxaliplatin and irinotecan in addition to 5-fluorouracil (5FU) or Capecitabine is used [9]. Targeted therapies, including monocolonal antibodies against VEGF (bevacizumab) or EGFR (cetuximab or panitumumab) are used in the metastatic setting in combination with chemotherapy depending on RAS mutational status and tumor location (left vs. right) [9,10]. In addition, immunotherapy has proven to be an effective treatment modality in the setting of microsatellite instability (MSI) high CRC [10]. For patients with unresectable lesions, treatment is a combination of maximum tumoral cytoreduction and chemotherapy [11]. Despite the advances in treatment, advanced disease remains to be associated with poor survival and resistance to cytotoxic and targeted chemotherapies is still occurring [12].

## 2. Repurposing Approved Drugs in Colon Cancer

Drug repurposing or repositioning involves using approved drugs for conditions different from their original indication [13,14,15]. Several drugs have acquired additional use in the past years and have been reintroduced into practice fueled by this phenomenon. For instance, thalidomide, discontinued from its original use as an antiemetic, is currently used for multiple myeloma [16] and moderate to severe erythema nodosum leprosum [17]. Another example is Sildenafil which preserves both its primary indication for erectile dysfunction [18] and repurposed indication as a treatment option for idiopathic pulmonary hypertension [19]. 

Drug repurposing has regained a significant role as a convenient, fast, and relatively safe drug development strategy. New drug development usually takes around 10–15 years on average [20], with a success rate reported from 2 to 10% [21,22]. According to the U.S. Food and Drug Administration (FDA), as of 2018, the compound percentage of drugs reaching stage 4 clinical trials was around 6% [23]. Drug repurposing offers significantly shorter development times and lower investments described as 160 million times lower, particularly costs regarding safety testing, molecular characterization, safety profiling, and initial marketing. It leverages known genetic, pharmacodynamic, pharmacokinetic, and adverse effect profiles, usually bypassing stage 1 clinical trials [24]. Therefore, this approach represents a more cost-efficient, expedited, and less risky strategy than traditional drug development [21]. 

Many successful reintroductions and alternative indications second repurposing as a feasible option in many areas of medicine. Aspirin, for example, has acquired a wide range of indications, ranging from acutely therapeutic to prolonged preventative ones [14,25]. The cardiovascular field further illustrates this diversity with the recent supportive evidence of SGLT-2 inhibitors, initially approved for hyperglycemia management, for heart failure management regardless of the patients’ ejection fraction and notwithstanding their diabetes status [26,27,28,29]. Therapeutics for Alzheimer’s disease have been highly reliant on this strategy. Since memantine in 2003, no new drugs had been approved until the FDA granted the recent fast track concession for aducanumab in 2021 [14,30]. As of 2017, approximately 27 FDA-approved drugs were being evaluated for Alzheimer’s disease in stages 1–3 clinical trials [14]. 

Oncology has also gained benefits from drug repurposing. Estimation is that 5% of the anticancer drugs entering phase 1 clinical trials are eventually approved [31]. Certain calcium channels blockers such as felodipine and amlodipine besylate undermine filopodia stability in cancer cells, decreasing the likelihood of progression, invasion, and metastasis [32]. Metformin, classically an antidiabetic drug, has been described to decrease tumor growth. Although metabolic reprogramming halting oxidative phosphorylation and multi-targeted mTOR inhibition have hypothesized metformin’s antitumoral activity, precise mechanisms remain obscure [21,33,34].

The benefits of drug repurposing are evident after their serendipitous discovery and raise interest in predictive tools to optimize outcomes. Many approaches group together into either experimental or computational models [24,35]. The former usually involves either in vitro analysis measuring affinity and interaction stability, also called binding assays, or combined in vitro/in vivo models using compound libraries to test for cellular lineage changes, known as the phenotypic model. The phenotypic approach aims to reproduce diseases in an experimental cellular environment and relies on known compound libraries to test and characterize cellular responses [24]. Alternatively, known compounds have been assessed using in silico models stemming from structure-based principles: direct molecular docking, inverse molecular docking, and receptor-based pharmacophore searching [36]. Drug-based strategies use established drug information such as pharmacodynamics, biochemical, adverse effect profiles, and genomic data to determine potential alternative uses. Transcriptomics data are especially valuable to depict deviant cellular responses to diverse pathologic states, notably those with solid genetic pathomechanisms. Conversely, knowledge-based strategies use well-characterized molecular disease mechanisms to depict candidates for drug repurposing [35].

Large genetic and disease datasets are becoming publicly available, and computational tools for processing massive data are evolving accordingly. Computational-based drug repurposing uses data mining, machine learning, and network analysis to distill large datasets involving disease-specific transcriptomics, proteomics, drug efficacy, responses, and even clinical variables [35]. This information provides insight into complex biologic processes such as epigenetic regulation in cancer cells. Furthermore, drug repurposing approaches may be used for epigenetic reprogramming of cancer cells to increase susceptibility via differential transcriptome expressions. A study characterized 45 FDA-approved drugs yielding synergistic activity with histone deacetylating agents and methylation inhibitors. Additionally, they characterized 85 FDA-approved medications that antagonized the action of these drug families, thwarting favorable responses in colon cancer cells. Altogether, these findings illustrate the benefits and complexity of drug repurposing to design personalized and highly effective treatment plans that account for previously unknown drug interactions [37] (Table 1).

### 2.1. Anti-Hypertensives and Anti-Arrhythmic Drugs

Angiotensin I converting enzyme inhibitors (ACEIs) and angiotensin II receptor blockers (ARBs) are commonly used drugs that have life-prolonging effects on patients treated for several diseases including but not limited to hypertension and heart failure [90]. An in vivo study by Kubota et al. suggested that both ACEIs and ARBs suppress colitis-induced CRC by decreasing chronic inflammation and oxidative stress in obese mice [38]. In another study by Kedika et al., patients who had one or more histologically confirmed adenomatous polyps on an index colonoscopy and received lisinopril—an ACEI—had a 41% reduction in the risk of developing similar polyps over the next 3–5 years [39].

Beta blockers (BB) are class II antiarrhythmic drugs used primarily to treat cardiovascular diseases and many other conditions [91]. In a study by Tapioles et al., Nebivolol was shown to selectively inhibit mitochondrial respiration in an HCT-116 colon cancer cell line by decreasing the activity of complex I of the respiratory chain and restraining the growth of colon cancer cells, hinting towards a repurposing potential for this drug in colon cancer [40]. Furthermore, Engineer et al. demonstrated that the combination of ACEI/ARB with BB was associated with increased survival, decreased hospitalization, and decreased tumor progression in advanced CRC [92].

### 2.2. Nonsteroidal Anti-Inflammatory Drugs

Nonsteroidal anti-inflammatory drugs (NSAIDs) employ their anti-inflammatory, analgesic, and antipyretic properties by inhibiting the cyclooxygenase (COX) enzymes [93]. COX-2 overexpression is a major risk factor for the development of CRC [94]. The therapeutic effect of aspirin in CRC can be explained by inhibition of COX-2 as well as the c-MYC transcription factor [41,42]. Furthermore, aspirin blocks platelet activity which is implicated in cancer metastasis and immune evasion [43]. However, Chan et al. argued that aspirin must be used for more than 10 years to achieve a statistically significant reduction in COX-2 positive cancer [44].

Celecoxib works by selectively and reversibly inhibiting COX-2, and thus acts to decrease inflammation and pain without affecting platelets [95]. Many studies concluded that celecoxib increases radiosensitization of CRC cells [96,97]. Celecoxib also affects p53 by regulating the expression of p21 and CyclinD1 in a COX-2-independent manner, by upregulating BCCIP, increasing radiosensitivity in the HCT116 CRC cell line [45]. A randomized controlled trial by Bertagnolli et al. showed that celecoxib was effective for the secondary prevention of colorectal adenomas and decreased the cumulative incidence of adenomas after 3 years from 60.7% in the placebo arm to 43.2% in patients receiving 200 mg of celecoxib twice daily [46].

### 2.3. Anti-Hyperlipidemic Drugs

Statins markedly inhibit HMG-CoA reductase, the enzyme that controls the rate-limiting step in the cholesterol synthesis pathway in the liver [98]. Remarkably, in a large study including 1953 patients with CRC and 2015 controls, the use of statins for at least 5 years was associated with a significantly reduced relative risk of developing CRC (odds ratio (OR) = 0.50; 95% confidence interval (CI), 0.40–0.63) [99]. In vivo, lovastatin was shown to restrict cancer progression and metastasis formation by inhibiting MACC1 [47]. In a large meta-analysis including a total of 31 studies and involving more than 1.6 million subjects, statins were shown to have a moderate protective effect against developing CRC [48].

### 2.4. Anti-Diabetic Drugs

Metformin, an oral anti-diabetic medication used for type 2 diabetes mellitus, is a biguanide drug that increases insulin sensitivity, decreases intestinal absorption of glucose, and decreases its production by the liver [100]. Previous studies have shown a protective effect of metformin in CRC risk and prognosis [101,102]. The current understanding is that metformin inhibits the mammalian target of rapamycin (mTOR) pathway which plays a central role in CRC cell growth and proliferation [49]. Furthermore, metformin downregulates IGF receptor activation through decreasing insulin and insulin growth factor, resulting in decreased proliferation in colorectal neoplasia [103,104]. Inhibition of mTOR is achieved through inhibition of mitochondrial mammalian respiratory chain complex I followed by activation of liver kinase B1 and downstream target Adenosine monophosphate-activated protein kinase (AMPK) [50,51]. Other research has shown that metformin, through modulation of oxidative stress and nuclear factor-κB (NF-κB) inflammatory responses would induce apoptosis in CRC cell lines [52,53]. Metformin may also increase sensitivity of cancer cell lines to chemotherapeutic agents such as 5-Fluorouracil, irinotecan, and paclitaxel [105,106,107]. 

Dapagliflozin, another oral antihyperglycemic medication used for type 2 diabetes mellitus works by inhibiting the sodium/glucose cotransporter 2 (SGLT2) in the proximal tubules of the kidney [108]. Dapagliflozin decreases the adhesion of CRC cells by affecting cellular interaction with Collagen types I and IV through activating ADAM10, which subsequently causes a loss in the full-length DDR1 [54]. DDR1 binding to Collagens I and IV is necessary to stimulate cell–collagen interactions [109]. Dapagliflozin also decreases colon cell proliferation by increasing Erk phosphorylation in the HCT116 human colon cancer cell line [55]. In a case report by Okada et al., SGLT2 inhibition in combination with the EGFR inhibitor, cetuximab, reduced both tumor size and carcinoembryonic antigen (CEA) levels in CRC with liver metastasis [110].

### 2.5. Anti-Helminthic Drugs

Mebendazole is a broad spectrum benzimidazole that inhibits microtubule synthesis by blocking tubulin polymerization [111]. Mebendazole has cytotoxic activity against different CRC cell lines such as HCT-116, RKO, HT-29, HT-8, and SW626 [56,112]. Nygren and Larsson reported that mebendazole induced remission of metastatic lesions in a patient with refractory metastatic CRC [113]. Another study carried out on mice with a constitutional mutation in the Adenomatous polyposis coli (*APC*) gene showed that the combination of mebendazole and sulindac (an NSAID) decreased both the number and size of intestinal microadenomas by inhibiting MYC and COX-2 pathways, angiogenesis, and the release of pro-tumorigenic cytokines [57].

Niclosamide is a salicylamide derivative that acts by uncoupling oxidative phosphorylation and regulating different signaling pathways [114]. Niclosamide downregulated the Wnt/β-catenin cascade, which is aberrantly activated in 80% of sporadic CRC [115], in both in vitro and in vivo studies [58] and resulted in decreased proliferation in multiple human CRC cell lines such as HCT-116, Caco2, and HT-29 [59], possibly via the induction of autophagy [116]. Furthermore, a recent study by Kang et al. demonstrated that niclosamide could be combined with metformin to synergistically inhibit *APC*-mutant CRC by suppressing Wnt and YAP [117].

### 2.6. Anti-Retroviral Drugs

Tenofovir is a nucleoside antiretroviral drug that acts by inhibiting the reverse transcriptase enzyme [118]. Tenofovir also inhibits the activity of human telomerase [119], a crucial enzyme for tumorigenesis and cancer proliferation, whose inhibition represents a promising therapeutic strategy in cancer treatment [120,121]. Sherif et al. demonstrated that rats receiving tenofovir at a dose of 50 mg/kg for 24 weeks had diminished colorectal cell proliferation attributed to decreased Bcl-2 and cyclin D1 expression [60]. Zidovudine, also known as azidothymidine, is another nucleoside reverse transcriptase inhibitor (NRTI) used in the treatment of human immunodeficiency virus (HIV) [122]. Brown et al. demonstrated Zidovudine’s telomerase inhibition activity in the HT-29 colon cancer cell line [61]. Furthermore, Fang et al. showed that the antitumor activity of zidovudine in colon cancer cells is mediated by increased expression of the p53-Puma/Bax/Noxa pathways favoring apoptosis, and activation of the p53-p21 pathway promoting cell cycle arrest [62]. Efavirenz is a non-nucleoside reverse transcriptase inhibitor (NNRTI) used in the treatment of HIV that is selectively cytotoxic to different tumor cell lines, including colorectal carcinoma, by activating the phosphorylation of p53 [63].

Protease inhibitors (PI) are also drugs that suppress the action of HIV proteases to inhibit viral growth, infectivity, and replication [123]. Indinavir and Saquinavir are PI that suppress the growth of human tumor cells by blocking angiogenesis and matrix metalloproteinases to inhibit tumor invasion and progression. [64]. Furthermore, Mühl et al. reported that Ritonavir synergizes with butyrate to induce apoptosis of CRC cells [66]. The anticancer effect of Ritonavir is most likely due to the inhibition of proteolytic degradation, which causes the accumulation of p21 [68], the decreased production of TNF-α, IL-6, IL-8, and VEGF [67], and the increased expression of anti-inflammatory heme oxygenase-1 [66].

Integrase inhibitors are the latest class of antiretroviral drugs which were approved for HIV therapy due to their efficacy, tolerability, and safety [124]. Raltegravir is an integrase inhibitor that inhibits the Fascin-1-dependent invasion of colorectal tumor cells in vitro and in vivo [69]. Fascin-1 is an actin cross-linking protein whose elevated expression is associated with aggressive clinical progression, dismal prognosis, increased recurrence, and worse survival outcomes in patients with CRC [125,126].

### 2.7. Anti-Microbials

Other anti-microbial drugs have been investigated for repurposing in colon cancer treatment including doxycycline, a semi-synthetic antibiotic derivative of tetracycline used in the treatment of a wide variety of infections [127]. Doxycycline has also been shown to inhibit matrix metalloproteinases [128]. Onoda et al. demonstrated that a combination therapy consisting of doxycycline and a COX-2 inhibitor suppressed colon cancer cell proliferation and invasion [71]. Doxycycline reportedly induced apoptosis in a dose-dependent manner through activation of caspases, release of cytochrome C, and translocation of Bax [70].

Another antibiotic with potential in cancer therapeutics is clarithromycin. Clarithromycin is a potent inhibitor of tumor-induced angiogenesis [73] showing increased efficacy when combined with approved anticancer drugs [72,75,129]. It is also implicated in attenuating autophagy in myeloma cells [130]. Targeting autophagy is considered a promising strategy for colon cancer therapy [131,132]. In a study by Petroni et al., clarithromycin was indeed shown to modulate autophagy in human CRC cells and inhibited the growth of tumors by targeting hERG1 [74].

The inhibition of autophagy as a mechanism of anticancer activity is also shared by azithromycin, another macrolide antibiotic [76,77]. Qiao et al. demonstrated that azithromycin had a synergistic antitumor activity with the tumor necrosis factor-related apoptosis-inducing ligand (TRAIL) in colon cancer cells. Azithromycin may also suppress autophagy by upregulating the expression of p62 and LC-3B to ultimately induce colon cancer cell death [78].

Gemifloxacin is a fluoroquinolone used in the setting of community-acquired pneumonia and acute exacerbations of chronic bronchitis [133]. Kan et al. demonstrated that gemifloxacin inhibits the migration and invasion of SW620 and LoVol colon cancer cells and downregulates Snail to reduce epithelial-to-mesenchymal transition (EMT). Gemifloxacin also suppresses the NF-κB pathway and cytokine-mediated cell migration and invasion as shown by decreased levels of tumor necrosis factor alpha (TNF-alpha), interleukin 6 (IL-6), IL-8, and vascular endothelial growth factor (VEGF) [79].

Antimalarials are also being considered for the treatment of colon cancer. Artesunate is an antimalarial agent recommended for the treatment of patients with severe Plasmodium falciparum malaria [134]. In a preclinical model of CRC, artesunate was found to suppress inflammation and oxidative stress [81]. Efferth et al. demonstrated a cytotoxic action of artesunate on tumor cells via both p53-dependent and -independent pathways [135] implicated in downregulation of β-catenin [80]. Mefloquine, another antimalarial drug, was found to induce growth arrest and apoptosis of CRC cells in mice via inhibition of the tumor NF-κB signaling pathway [82].

### 2.8. Others

Drugs used for neurological conditions are increasingly being considered as therapeutic options in cancer patients [136,137]. Friedmann et al. demonstrated that valproate, a histone deacetylase inhibitor (HDACi), dose-dependently reduced the viability of adenocarcinoma cell lines, particularly when combined with mitomycin C [138]. Additionally, in a study by Mologni et al., valproate was found to enhance bosutinib cytotoxicity in colon cancer cells [139]. This may be explained by increasing histone hyperacetylation of H3 and H4 to enhance antitumor activity and by relieving HDAC-driven transcriptional repression [83,140].

Fluoxetine is a selective serotonin reuptake inhibitor (SSRI) that is used in the treatment of major depressive disorder [141]. In a study examining murine colitis-associated colon cancer, fluoxetine was found to inhibit NF-κB activation and decrease TNF-α-mediated IκB kinase (IKK) and IκBα phosphorylation; thus suppressing dextran sulfate sodium (DSS)-induced colitis and colitis-associated tumorigenesis [84]. Furthermore, Kannen et al. studied the antiproliferative effects of fluoxetine on HT29 colon cancer cells. Fluoxetine increased the percentage of HT29 cells in the G0/G1 phase of the cell cycle and enhanced the expression of p27 protein. Fluoxetine also suppressed the development of dysplasia and vascularization-related dysplasia in colon tissue, and reduced VEGF expression and the number of cells with angiogenic potential, such as CD133, CD34, and CD31-positive cell clusters [85]. 

Sirolimus, also known as Rapamycin, is an FDA-approved mTOR inhibitor used in the prophylaxis of renal graft rejection [142]. Mussin et al. demonstrated that a combination of sirolimus and metformin synergistically inhibits colon tumor growth both in vitro and in vivo [86]. He et al. revealed that mTOR inhibitors induce apoptosis in colon cancer cells via CHOP-dependent DR5 induction of 4E-BP1 dephosphorylation resulting in decreased tumor proliferation, angiogenesis, and invasion [88]. In addition, Wang et al. demonstrated that sirolimus suppresses the *FBXW7*-loss-driven EMT through its mTOR inhibition activity, thereby decreasing CRC cell migration and invasion [87].

Finally, it is worth noting that probiotics have also been considered for the treatment of CRC. For instance, Hu et al. found that butyrate decreased the transcription of the pro-proliferative miR-92a in human CRC cells [89]. Furthermore, butyrate can also induce apoptosis of DLD-1 colon cancer cells by synergizing with ritonavir [66].

## 3. Clinical Trials on Drug Repurposing in Colon Cancer

**Figure 1:**
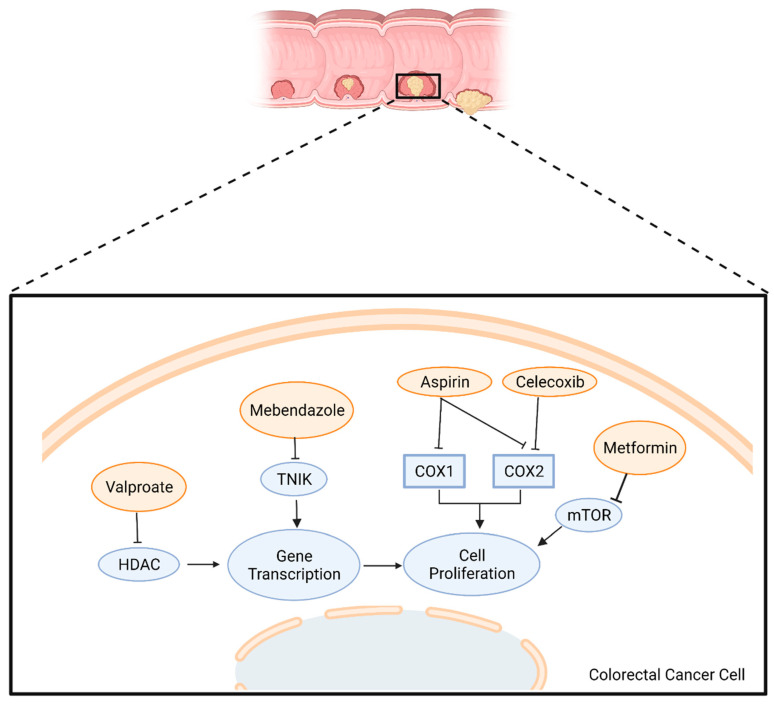
Mode of action of candidate repurposed drugs being tested in clinical trials for patients with CRC. HDAC: Histone Deacetylase; TNIK: TRAF2 And NCK Interacting Kinase; COX: Cyclo-oxygenase; mTOR: mammalian Target of Rapamycin. Adapted from “Round-Cell Background”, by BioRender.com (2022). Retrieved from https://app.biorender.com/biorender-templates accessed on 15 April 2022.

Despite the heterogeneity of potential drugs that can be repurposed, only a few collectively form the most promising treatments for CRC and have been incorporated in clinical trials. A brief overview of the mechanism of action of these drugs is summarized in Figure 1.

Aspirin is the most represented drug in current clinical trials investigating candidate drugs for repurposing in the treatment of CRC (Table 2). One phase 3 clinical trial involves the use of aspirin as an adjuvant component in stages II and III *PIK3CA*-mutated colon cancer patients. The trial aims to determine whether the daily consumption of 100 mg of aspirin is effective in reducing recurrence and improving survival compared to placebo (ClinicalTrials.gov; NCT02467582). Two similar phase 3 clinical trials aim to determine the effect of 80 mg daily adjuvant aspirin on survival in stages II and III colon cancer patients compared to placebo (ClinicalTrials.gov; NCT02301286 and NCT03464305). ASPIK French is another phase 3 clinical trial whose goal is to determine local or distant recurrence or second CRC or death from any cause, whichever occurs first, in patients with surgically resected PI3K-mutated colon cancer taking 100 mg of daily aspirin as compared to placebo (ClinicalTrials.gov; NCT02945033). Finally, the ASCOLT phase 3 clinical trial is studying the 5-year disease-free and overall survival in patients with Dukes C or high-risk Dukes B CRC taking 200 mg daily aspirin for 3 years (ClinicalTrials.gov; NCT00565708).

NSAIDs are also among the drugs currently under study for repurposing in CRC therapeutics. For instance, the NICHE phase 2 clinical trial involves the administration of celecoxib along with nivolumab and ipilimumab for stages I to III colon cancer in the neoadjuvant setting. The adverse effects will be assessed to determine the regimen’s safety (ClinicalTrials.gov; NCT03026140).

Mebendazole, an anti-helminthic drug, is being tested in a phase 3 clinical trial for its use as an adjuvant component to FOLFOX with Bevacizumab in stage IV CRC patients (ClinicalTrials.gov; NCT03925662). Furthermore, MECORA is a phase 2 clinical trial which aims to examine the effect of metformin in non-diabetic patients with colon cancer where metformin will be administered before and after colon cancer surgery (ClinicalTrials.gov; NCT03359681).

A phase IB clinical trial aims to assess the safety of the antimalarial hydroxychloroquine along with Axitinib and hepatic chemoembolization in subjects with liver dominant metastatic CRC (ClinicalTrials.gov; NCT04873895). Finally, a dose-finding phase 1/2 clinical trial is studying the safety and recommended phase 2 dose (RP2D) of the combination of neratinib and sodium valproate in patients with advanced solid tumors, including RAS-mutated CRC patients (ClinicalTrials.gov; NCT03919292).

## 4. Conclusions and Future Directions

Despite the advances in oncology, cancer continues to be one of the leading causes of morbidity and mortality worldwide. CRC has the third highest mortality rate of all cancers, with poor survival rates in many groups. Moreover, CRC cases are expected to increase dramatically in the following decades [3]; this is why finding effective treatments for these patients is crucial. Developing new drugs and translating them into clinical practice from phases 1 to 3 is very long and expensive, with only 5% of oncology drugs resulting in FDA approval [33]. For this reason, drug repurposing has gained more interest as a fast and safe way to achieve better outcomes since the pharmacokinetic, pharmacodynamic, and toxicity profiles of these drugs are already established. The investigation of previously approved drugs with other indications for CRC treatment should identify new effective therapies with lower costs and shorter timelines. Many FDA-approved drugs for infectious, cardiovascular, metabolic, and other diseases are currently studied as possible cancer therapies. 

It is important to highlight that the heterogeneity of cancer physiology and response to therapy and the multiple resistance mechanisms these cells develop represent an enormous challenge in oncology. The combination of computational frameworks (translational bioinformatics, computational intelligence, and methodological and systems biology) in multidisciplinary teams has successfully sped up clinical trials for repurposing drugs [143]. The emergence of new technologies in this field, such as large-scale multi-omics sequencing, genome-wide positioning systems network (GPSnet) algorithms, and other artificial intelligence algorithms, has helped identify new targets for older drugs [144].

The tools have matured, and novel technological advancements have enabled the identification of drugs that are suitable for repurposing. Pushpakom et al. elegantly highlighted the recent ongoing drug repurposing strategies [145]. In the era of big data, computational-based approaches will be a driving force in this field. These strategies range from mapping drug-binding sites and implicated downstream pathways to developing correlative signatures of transcriptomic and genetic data and their integration with clinical databases [145]. These approaches can help narrow down the list of candidate drugs that can be used for repurposing [146]. Nevertheless, experimental strategies relying on large-scale drug testing using in vitro and in vivo models remain to be an essential bridge to early phase clinical trials [145,147].

However, there are several limitations that might have rendered efforts in drug repurposing unsuccessful. Currently available drugs are approved at specific dosages to treat specific conditions. It is unknown whether these drugs are effective in treating other conditions such as CRC and whether different pharmacodynamic and pharmacokinetic properties are required for their activity in this setting [148]. Clinical trials are crucial to answer these questions; however, funding efforts have remained to be important obstacles as well as regulatory and organizational hurdles [149]. Despite that, not all trials translate into a clinical benefit which has recently been observed during the COVID-19 pandemic where a lot of negative trials have been reported [150]. This may be due to poorly designed trials or due to the fact that in vitro effects observed are simply not always translated into clinical benefit in larger groups of patients [150,151].

In conclusion, the overall impact of repurposing drugs must be studied in terms of survival and other aspects such as toxicity and side effects. Although the success rate is not so high, this is a promising strategy that must be deeply studied to provide new therapies for patients with CRC. Drugs that are currently being tested in clinical trials including but not limited to metformin, mebendazole, aspirin, celecoxib, and valproic acid are promising candidate drugs that might have substantial benefit in the CRC clinic.

## Figures and Tables

**Table 1:** Summary table of the drugs that have been repurposed to be used in colon cancer.

Reference	Drug	Original Indication	Possible Mode(s) of Action	Effect(s)
[38,39]	ACEIs/ARBs	Hypertension	Decreased chronic inflammation and oxidative stress	Reduced risk of adenomatous colon polyps
[40]	Nebivolol	Hypertension and other indications	Inhibition of mitochondrial respiration by decreasing the activity of Complex I of the respiratory chain	Suppressed the growth of colon cancer cells
[41,42,43,44]	Aspirin	Antiplatelet	Inhibition of COX-2, c-MYC transcription factor, and the antiplatelet mechanism of action	Decreased cancer metastasis and immune evasion
[45,46]	Celecoxib	Anti-inflammatory	Effect on p53 by regulating the expression of p21 and CyclinD1 in a COX-2-independent manner Upregulation of BCCIP Increased radiosensitivity in HCT116 cell line	Decreased incidence of adenomatous polyps.
[47,48]	Lovastatin	Antilipidemic	Inhibition of MACC1	Restricted cancer progression and metastasis formation
[49,50,51,52,53]	Metformin	Antihyperglycemic	Inhibition of mTOR Modulation of oxidative stress and nuclear factor-κB inflammatory responses	Apoptosis in CRC cell lines
[54,55]	Dapagliflozin	Antihyperglycemic	Effect on cellular interaction with Collagen types I and IV Increased Erk phosphorylation	Decreased adhesion and proliferation of colon cancer cells
[56,57]	Mebendazole	Anti-helminthic	Inhibition of MYC	Cytotoxic activity against different colon cancer cell lines
[58,59]	Niclosamide	Anti-helminthic	Downregulation of the Wnt/β-catenin cascade	Decreased proliferation in multiple human CRC cell lines
[60]	Tenofovir	Anti-retroviral (anti-HIV drug)	Decreased Bcl-2 and cyclin D1 expression	Inhibition of proliferation, oxidative stress, and inflammation
[61,62]	Zidovudine	Anti-retroviral (anti-HIV drug)	Increased expression of the p53-Puma/Bax/Noxa pathways Activation of the p53-p21 pathway	Apoptosis Cell cycle arrest
[63]	Efavirenz	Anti-retroviral (anti-HIV drug)	Activation of the phosphorylation of p53	Cytotoxic activity against different colon cancer cell lines
[64]	Indinavir	Anti-retroviral (anti-HIV drug)	Proteasome-independent block of angiogenesis and matrix metalloproteinases	Suppressed growth
[64,65]	Saquinavir	Anti-retroviral (anti-HIV drug)	Proteasome-independent block of angiogenesis and matrix metalloproteinases Inhibition of proteolytic degradation and accumulation of p21	Apoptosis Suppressed growth
[66,67,68]	Ritonavir	Anti-retroviral (anti-HIV drug)	Inhibition proteolytic degradation and accumulation of p21 Decreased production of TNF-α, IL-6, IL-8, and VEGF Increased expression of heme oxygenase-1	Apoptosis Suppressed angiogenesis
[69]	Raltegravir	Anti-retroviral (anti-HIV drug)	Blockage of fascin-1	Suppressed invasion
[70,71]	Doxycycline	Antibiotic	Inhibition of matrix metalloproteinases Activation of caspase-3, -8, and -9 Release of cytochrome c and Bax translocation	Apoptosis Suppressed proliferation and invasive potential
[72,73,74,75]	Clarithromycin	Antibiotic	Inhibition of autophagy by targeting hERG1	Suppressed angiogenesis Suppressed growth of colon cancer cells
[76,77,78]	Azithromycin	Antibiotic	Inhibition of autophagy by upregulating p62 and LC-3B	Apoptosis
[79]	Gemifloxacin	Antibiotic	Inhibition of NF-κB activation Inhibition of TNF-α, IL-6, IL-8, and VEGF	Suppressed cell migration and invasion
[80,81]	Artesunate	Antimalarial	Downregulation of β-catenin	Apoptosis Cytotoxicity
[82]	Mefloquine	Antimalarial	Inhibition of NF-κB activation	Apoptosis Growth arrest
[83]	Valproate	Antipsychotic	Histone hyperacetylation Relief of HDAC-mediated transcriptional repression	Reduced viability Enhanced cytotoxicity
[84,85]	Fluoxetine	Antidepressant	Inhibition of NF-κB activation and IKK phosphorylation Cell-cycle arrest at G0/G1 Enhanced p27 expression Reduced VEGF expression	Suppressed colitis-associated tumorigenesis Suppressed dysplasia and angiogenesis
[86,87,88]	Sirolimus	Prevention of kidney transplant rejection	CHOP-dependent DR5 induction on 4E-BP1 dephosphorylation Suppressed FBXW7 loss-driven EMT	Apoptosis Decreased angiogenesis Suppressed proliferation and invasion of colon cancer cells
[89]	Butyrate	Probiotic	Inhibition of miR-92a	Suppressed proliferation of colon cancer cells

**Table 2:** Examples of some repurposed drugs currently being clinically investigated for the treatment of colon cancer.

Clinical Trial Number	Phase	Status	Estimated Completion Date	Intervention/Treatment	Patient Population	Patients Enrolled	Primary Outcome Measures	Secondary Outcome Measures
NCT02467582	3	Active, not recruiting	June 2029	Aspirin	Stages II and III *PIK3CA*-mutated CRC previously treated with surgery	185	DFS after 6 years	Time to recurrenceOS Cancer-specific survival Adverse events
NCT02301286	3	Recruiting	September 2022	Aspirin	Stages II and III CRC	1588	OS	DFS TTF
NCT03464305	3	Recruiting	December 2026	Aspirin	Stages II and III CRC	400	5-year OS	DFS TTF
NCT02945033	3	Recruiting	July 2024	Aspirin	*PI3K*-mutated CRC	246	Recurrence or second CRC or death, whichever occurs first	5-year OS Adverse events
NCT00565708	3	Active, not recruiting	June 2026	Aspirin	Dukes C and high-risk Dukes B CRCs	1587	DFS	OS
NCT03026140	2	Recruiting	January 2022	Nivolumab + Ipilimumab with or without Celecoxib	Stages I to III CRC	60	Incidence of adverse events	Immune activating capacity of immunotherapyRelapse-free survival
NCT03925662	3	Recruiting	December 2028	FOLFOX + bevacizumab with or without mebendazole	Stage IV CRC	40	ORR	-
NCT03359681	2	Recruiting	January 2022	Metformin	CRC	48	Ki67 expression on tumor samples	Cleaved Caspase-3 expression Immunoscore Immunological changes in blood samples In vitro cell growth
NCT04873895	1	Recruiting	November 2023	Axitinib + hydroxychloroquine	Liver-dominant metastatic CRC	25	Serious adverse events	ORR in setting of liver metastasis PFS OS
NCT03919292	1/2	Recruiting	January 2024	Neratinib + valproate	Advanced solid tumors including CRC	113	Recommended phase 2 dose	Adverse events Antitumor effects PFS

Abbreviations: CRC: colorectal cancer; DFS: disease-free survival; ORR: objective response rate; OS: overall survival; PFS: progression-free survival; TTF: time-to-treatment-failure; FOLFOX: folinic acid + fluorouracil + oxaliplatin.
